# Alterations in Gastric Microbial Communities Are Associated with Risk of Gastric Cancer in a Korean Population: A Case-Control Study

**DOI:** 10.3390/cancers12092619

**Published:** 2020-09-14

**Authors:** Madhawa Gunathilake, Jeonghee Lee, Il Ju Choi, Young-Il Kim, Jaekyung Yoon, Woo Jun Sul, Jihyun F. Kim, Jeongseon Kim

**Affiliations:** 1 Department of Cancer Control and Population Health, Graduate School of Cancer Science and Policy, Goyang-si 10408, Gyeonggi-do, Korea; 1806104@ncc.re.kr; 2 Department of Cancer Biomedical Science, Graduate School of Cancer Science and Policy, Goyang-si 10408, Gyeonggi-do, Korea; jeonghee@ncc.re.kr; 3 Center for Gastric Cancer, National Cancer Center Hospital, National Cancer Center, Goyang-si 10408, Gyeonggi-do, Korea; cij1224@ncc.re.kr (I.J.C.); 11996@ncc.re.kr (Y.-I.K.); 4 Department of Systems Biology, Divisions of Life Sciences, and Institute Life Science and Biotechnology, Yonsei University, 50 Yonsei-ro, Seodaemun-gu, Seoul 03722, Korea; ymicrobes@yonsei.ac.kr (J.Y.); jfk1@yonsei.ac.kr (J.F.K.); 5 Department of Systems Biotechnology, College of Biotechnology and Natural Resources, Chung-Ang University, Anseong 17546, Gyeonggi-do, Korea; sulwj@cau.ac.kr

**Keywords:** gastric cancer, gastric microbiome, microbial dysbiosis index, pathways, case-control

## Abstract

**Simple Summary:**

The gastric microbial community has been identified as a specific risk factor for the gastric cancer (GC) risk in recent molecular epidemiology studies. The equilibrium of the gastric microbial community and their functions are very important to keep a proper gastric related health. However, dysbiosis where there is an imbalance of the microbiome in gastric environment leads to several pathological conditions including GC. Thus, understanding how alterations in gastric microbial communities are associated with GC risk in large population-based studies is needed to implement possible preventive and curative strategies in the future. We derived a microbial dysbiosis index to observe the association with GC risk. Further, we predicted the microbial functions that are associated with GC risk. The findings of our study are important to understand certain pathogenic bacteria and their functions associated with GC risk. It might be helpful to develop novel preventive guidelines to prevent GC risk.

**Abstract:**

Although the microbiome has a potential role in gastric cancer (GC), little is known about microbial dysbiosis and its functions. This study aimed to observe the associations between the alterations in gastric microbial communities and GC risk. The study participants included 268 GC patients and 288 controls. The 16S rRNA gene sequencing was performed to characterize the microbiome. *Streptococcus_NCVM* and *Prevotella melaninogenica* species were highly enriched in cases and controls, respectively. Those who were in the third tertile of *P. melaninogenica* showed a significantly decreased risk of GC in total (odds ratio (OR): 0.91, 95% confidence interval (CI): 0.38–0.96, *p*-trend = 0.071). Class *Bacilli* was phylogenetically enriched in cases, while phylum *Actinobacteria*, class *Actinobacteria* were related to the controls. The microbial dysbiosis index (MDI) was significantly higher for the cases compared with the healthy controls in the female population (*p* = 0.002). Females in the third tertile of the MDI showed a significantly increased risk of GC (OR: 2.66, 95% CI: 1.19-5.99, *p*-trend = 0.017). Secondary bile acid synthesis and biosynthesis of ansamycins pathways were highly abundant in cases and controls, respectively. Dysbiosis of gastric microbial communities is associated with an increased risk of GC specifically in females.

## 1. Introduction

According to the GLOBOCAN estimates in 2018, gastric cancer (GC) has been identified as the fifth most common cancer type and is one of the main causes of cancer-related death in the world [1]. The GC incidence in eastern Asia, including Korea, is the highest worldwide—over four times higher than the rates in Western Europe [2]. In 2017, according to the Korea Central Cancer Registry (KCCR), the age-adjusted incidence rate of GC was 32.0 per 100,000 in the overall population, 46.4 per 100,000 in men, and 19.6 per 100,000 in women [3]. Various epidemiological studies have demonstrated that smoking [4,5,6,7], alcohol consumption [4,5,8], obesity and physical inactivity [4,5,9], family history of GC [10,11,12,13,14], and numerous dietary factors such as foods preserved with salts, pickled vegetables, low fruit and vegetable consumption, low dietary vitamin C and carotenoid intake, high salt consumption, high processed meat consumption, and high salt intake [4,5,15,16] are associated with an increased risk of GC. In addition, some of recent epidemiological studies have suggested the involvement of the gastric microbiome in GC occurrence by the induction of chronic inflammation or down regulation of host immunity [17,18,19,20,21,22,23].

In the gastric environment, innumerable bacteria form a complex and stable bacterial community that eventually plays an important role in the host mucosal immune response, energy and nutrient metabolism, pathogen elimination, and the development of several diseases including GC [24,25,26,27,28,29]. *Helicobacter pylori* (HP) infection plays a crucial role in the initial steps of GC carcinogenesis by enhancing inflammation [30]. It is widely implicated that GC carcinogenesis can occur because of the imbalance of the gastric microbial community [31]. Such an imbalance of the gastric microbial community is known as dysbiosis, which eventually leads to pathological conditions such as GC [22]. A dysbiosis condition with an imbalance of the microbial community in the stomach can lead to the formation of inflammatory biomarkers that can stimulate the carcinogenesis process [32,33]. Moreover, bacteria that are prominent during gut dysbiosis can secrete toxins able to interfere with host cell growth, finally predisposing the host organism to cancer development [34].

The microbiome is involved in metabolism and communicates with the host to proceed with physiological functions. It releases several metabolites that are essential for our bodily functions [35]. It can be noted that there is a two-way communication between gut microbes and the human immune system [35]. Emphasizing the metabolic functions associated with gut microbiota is important because several possible microbial mechanisms are involved in carcinogenesis through microbiota. Dysbiosis helps to increase the pH and innate immune response that generates changes in the acquisition of nutrients, metabolism, and the survival of members of the normal microbiota; however, the expansion and development of other species, such as *Clostridium colicanis*, *Lactobacillus gasseri*, *Lactobacillus reuteri*, *Peptostrptococcus stomatis*, and *Bacteroides fragilis* promote carcinogenesis [36]. Thus, understanding how dysbiosis influences metabolic reactions and inflammatory responses is critical.

In this study, we hypothesized that the alterations in gastric microbial communities are associated with risk of GC. We conducted a case-control study to apply compositional analysis of microbiome data to derive a microbial dysbiosis index (MDI) associated with GC risk and further identify the microbial abundances and enriched metabolic functions associated with subsequent GC risk.

## 2. Results

### 2.1. General Characteristics

The general characteristics of the study participants are presented in the Supplementary File (Table S1). In brief, GC cases had a higher proportion of current smokers (29.1%) than the controls (17.7%). Higher proportions of GC cases had a family history of GC (*p* = 0.003), had not engaged in regular exercise (*p* < 0.001), had lower education (*p* < 0.001), had lower employment rates (*p* = 0.037), had low levels of monthly income (*p* < 0.001), and had an HP-positive status (*p* < 0.001) than the controls. Daily energy consumption was higher in GC cases than the controls (*p* < 0.001).

### 2.2. Microbial Taxa Distribution and Microbial Diversity

At the species level, HP was the most dominant species in GC cases and controls (91.57 ± 18.56 and 87.93 ± 27.18%; *p* = 0.065), followed by *Haemophilus parahaemolyticus* (0.88 ± 3.61 and 0.88 ± 3.08%; *p* = 0.987), *Streptococcus_NCVM* (0.78 ± 2.45 and 0.75 ± 2.87%; *p* = 0.904), *Neisseria subflava* (0.49 ± 1.49 and 0.66 ± 2.56%; *p* = 0.356), *Neisseria perflava* (0.47 ± 1.85 and 1.05 ± 4.61%; *p* = 0.048), *Haemophilus_JUTE* (0.37±0.84 and 0.51 ± 1.26%; *p* = 0.113), *Prevotella melaninogenica* (0.32 ± 1.00 and 0.71 ± 2.21%; *p* = 0.007), *Staphylococcus warneri* (0.23 ± 3.49% and 0.09 ± 0.001%; *p* = 0.290), *Prevotella pallens* (0.20 ± 0.80% and 0.40 ± 1.45%; *p* = 0.044), *Streptococcus_PAC001345* (0.20 ± 0.66% and 0.28 ± 1.06%; p = 0.274), and *Veillonella atypica* (0.19 ± 0.80% and 0.31 ± 1.15%; *p* = 0.148) (Figure S1).

Table 1 shows the alpha diversity indices between cases and controls. There was a significantly higher Shannon index in the controls than GC cases (*p* = 0.030). A significantly higher Shannon index was observed in controls than GC cases in females (*p* = 0.007). There was a significantly higher richness in GC cases compared with controls in the total population (*p* = 0.009) and males (*p* < 0.001). Pilou evenness based on the Shannon index was significantly higher in controls than GC cases in the total population (*p* = 0.004) and in females (*p* = 0.004).

### 2.3. Bacterial Community Structure

**Figure 1:**
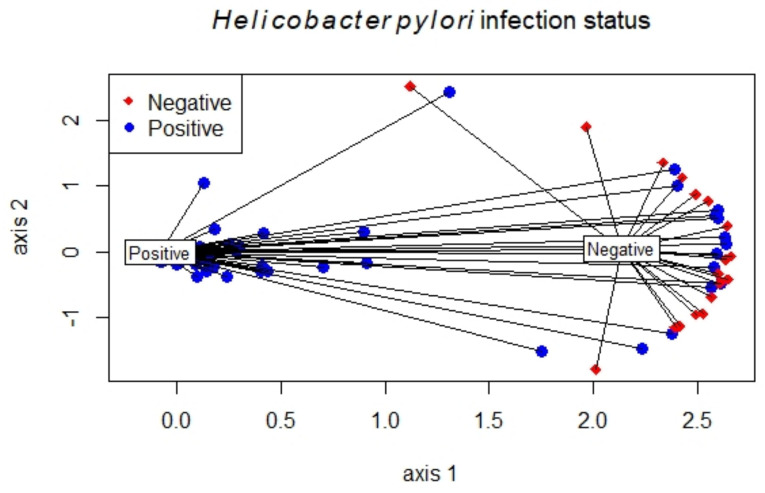
Nonmetric multidimensional scaling (NMDS) for microbial community composition based on *Helicobacter pylori* (HP) infection status.

To analyze differences in microbial community structure between groups, we assessed the beta diversity (Figure S2). The overall difference was visualized using a principal coordinate analysis (PCoA) plot. The diversity described in the PCoA plot by the top two principal coordinates was 32.4% based on Bray–Curtis distance. The composition of the microbiota of patients with GC was significantly different from the healty controls (analysis of similarities (ANOSIM) R = −0.00015, *p* = 0.004). Nonmetric multidimensional scaling (NMDS) was performed to relate the microbial composition with the metadata of the study population. The multidimensional space of the microbial species level indicated an excellent representation in reduced dimensions with a stress value of 0.043. The nonmetric correlation coefficient between observed dissimilarity and ordination distance was (R^2^ = 0.998). According to the NMDS results, the microbial composition of the HP-positive and -negative groups was significantly different (Permutational Multivariate Analysis of Variance (PERMANOVA); *p* = 0.001) (Figure 1).

### 2.4. Differentially Abundant Taxa

**Figure 2:**
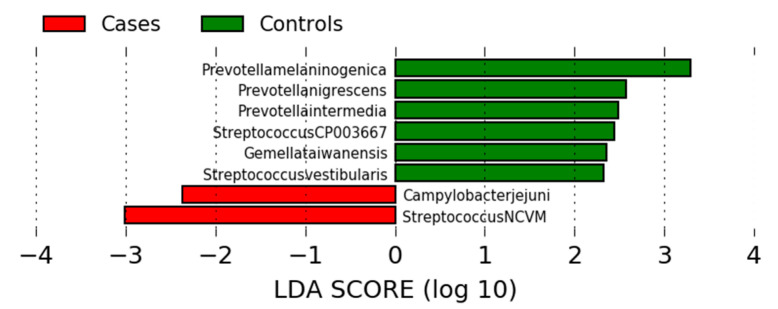
Linear discriminant analysis (LDA) of effect size (LEfSe) analysis plot for taxonomic species.

To identify the specific bacterial taxa associated with GC, different taxonomy levels were compared using linear discriminant analysis (LDA) and effect size (LEfSe) analysis based on the non-parametric factorial Kruskal–Wallis (KW) sum rank test to detect bacterial taxa with significant differential abundances between GC cases and controls. LEfSe then uses LDA to estimate the effect size of each differentially abundant taxa with the criteria of LDA ≥ 2 and *p* < 0.05. At the species level, eight species were differentially abundant in GC cases and controls among the total of 945 species. Of them, *P. melaninogenica, Prevotella nigrescens, Prevotella intermedia, Streptococcus_CP003667, Gemella taiwanensis,* and *Streptococcus vestibularis* were highly enriched in controls, whereas *Campylobacter jejuni,* and *Streptococcus_NCVM* species were highly abundant in GC cases (Figure 2).

### 2.5. Association between Candidate Taxa and GC Risk

Table 2 shows the association between the relative abundance of bacteria species and GC risk. Those in the third tertile of *P. melaninogenica* showed a significantly decreased risk of GC compared with the lowest tertile in total population (odds ratio (OR): 0.91, 95% confident interval (CI): 0.38–0.96, *p*-trend = 0.071). Subjects carrying *P. nigrescens* showed a significantly decreased risk of GC compared with those who did not (OR: 0.64, 95% CI: 0.43-0.94). In contrast, those carrying *S. vestibularis* showed a significantly increased risk of GC (OR: 2.41, 95% CI: 1.63–3.56) in the total population.

### 2.6. Phylogenetic Relationships

**Figure 3:**
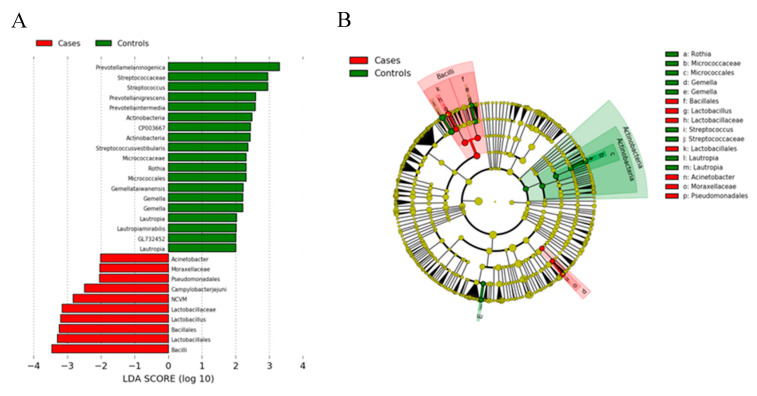
(**A**) Linear discriminant analysis of effect size (LEfSe) analysis plot of differentially abundant gastric microbial taxa between GC and control groups. (**B**) Cladogram representation of gastric microbiome taxa associated with GC based on the Ezbio database.

Figure 3A represents a histogram of the LDA scores calculated for the taxa showing the significant bacterial difference between GC cases and healthy controls. Figure 3B shows a cladogram based on the Ezbio database. The LDA score at the log_10_ scale is indicated at the bottom. The greater the LDA score, the more significant the microbial biomarker is in the comparison. The cladogram uses the LEfSe method, and indicates the phylogenetic distribution of gastric microbes associated with patients with GC (red indicates phylotypes statistically over-represented in GC) and in healthy subjects (green indicates phylotypes over-represented in healthy subjects). Each filled circle in the cladogram represents one phylotype; the phylum and class are indicated in their names on the cladogram, while the order, family, or genera are listed in the right panel. Taxa related to phylum *Actinobacteria*; class *Actinobacteria* were phylogenetically enriched in healthy controls, whereas taxa related to class *Bacilli* were phylogenetically enriched in GC cases.

### 2.7. MDI

**Figure 4:**
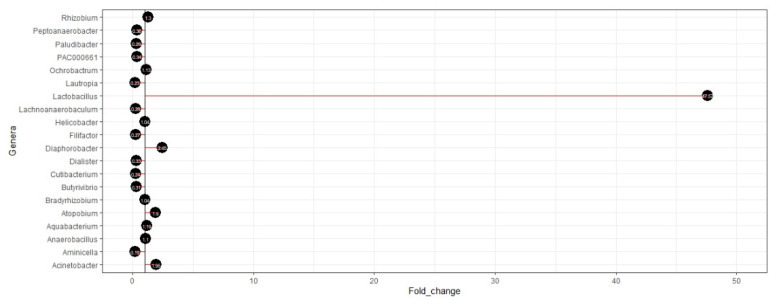
Diverging lollipop chart for differences in abundances for genera that were detected using compositionality corrected by renormalization and permutation (CCREPE). The fold change for each genus was calculated by dividing the mean abundance in cases by that of the controls.

The MDI was derived for the current study population based on a compositional data analysis of the microbiome using the compositionality corrected by renormalization and permutation (CCREPE) method. After selecting 64 of 73 genera based on the CCREPE method, fold change values were calculated to identify the genera that were increased in GC and decreased in GC. Of them, 13 genera were identified as increased in GC (fold change >1) (Table S2), while 51 genera were identified as decreased in GC (fold change <1) (Table S3). The top 10 genera that were diverged from the segment line to the right side (>1) and the top 10 genera that were diverged from the segment line to the left side (<1) are represented as a diverging lollipop chart for simplicity (Figure 4).

The MDI was higher for the GC cases compared with the healthy controls, and the result was marginally significant (*p* = 0.097) for the total population. A significantly higher MDI was observed for the GC cases than the controls in the female population (*p* = 0.002) (Table 3). Table 4 shows the association between MDI and GC risk for the total population, male and female. Even though the subjects in the third tertile of the MDI showed an increased risk of GC, the associations were not significant for the total and male populations. However, there was a significantly increased risk of GC for females with a higher MDI (OR: 2.66, 95% CI: 1.19–5.99, *p*-trend = 0.017) in model II. Table S4 shows the stratified analysis of the association between MDI and GC based on lifestyle factors. Among those who were never smokers, a significantly increased risk of GC was observed for the subjects in the third tertile of MDI in model II (OR: 2.37, 95% CI: 1.14–4.92, *p*-trend = 0.019).

### 2.8. Microbial Prediction Functions

**Figure 5:**
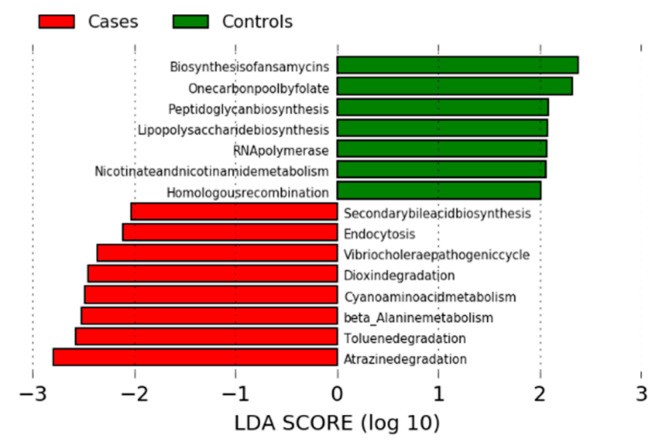
Comparison of microbial functional pathways between cases and controls.

LEfSe analysis was performed to distinguish the Kyoto Encyclopedia of Genes and Genomes (KEGG) pathways between GC cases and controls. Fourteen pathways presented significant difference between GC cases and control groups (Figure 5). Seven pathways were differentially abundant in the healthy controls whereas eight pathways were differentially abundant in GC cases (LDA score > 2 at 5% significance). Biosynthesis of ansamycins pathway was identified as one of the critical bacterial metabolic functions that was highly enriched in healthy controls.

The KEGG pathway of biosynthesis of ansamycins (ko01051, *p* < 0.001), one carbon pool by folate (ko00670, *p* < 0.001), peptidoglycan biosynthesis (ko00550, *p* < 0.001), lipopolysaccharide biosynthesis (ko00540, *p* = 0.028), RNA polymerase (ko03020, *p* < 0.001), nicotinate and nicotinamide metabolism (ko00760, *p* < 0.001), and homologous recombination (ko03440, *p* < 0.001) were enriched in healthy controls, whereas secondary bile acid biosynthesis (ko00121, *p* < 0.001), endocytosis (ko04144, *p* = 0.019), *Vibrio cholera* pathogenic cycle (ko05111, *p* < 0.001), dioxin degradation (ko00621, p<0.001), cyanoamino acid metabolism (ko00460, *p* < 0.001), beta-alanine metabolism (ko00410, *p* = 0.002), toluene degradation (ko00623, *p* = 0.012), and atrazine degradation (ko00791, *p* = 0.007) were abundant in GC cases (Table S5). Several metabolites that were involved in the KEGG pathways were identified based on the KEGG orthology (Table S5). Particularly, transketolase enzyme (K00615, *p* = 0.004), which is involved in the biosynthesis of ansamycins (ko01051) pathway was significantly enriched in healthy controls compared with GC cases. Formamidase (K01455, *p* < 0.001) and L-asparaginase (K13051, *p* = 0.033) enzymes, which are involved in the cyanoamino acid metabolism (ko00460) pathway, were highly enriched in GC cases compared with controls. Secondary bile acid biosynthesis (ko00121) pathway exhibited choloylglycine hydrolase (K01442) orthology, which was highly enriched in GC cases compared with the controls.

## 3. Discussion

In this study, the gastric microbiota composition was characterized between GC cases and controls. Calculation of the MDI by applying compositional analysis of microbiome data was used as a novel approach to identify gastric dysbiosis and further observe the associations with GC risk. Moreover, metagenomics functions were predicted to identify the pathways associated with GC risk. The NMDS results indicated that the microbial composition structures of HP-positive and -negative groups were significantly different (PERMANOVA; *p* = 0.001). The MDI was higher for GC cases than healthy controls for the total population. A significantly higher MDI was observed for GC cases than controls in the female population (*p* = 0.002). In females, those who were in the third tertile of the MDI showed a significantly increased risk of GC (OR: 2.66, 95% CI: 1.19-5.99, *p*-trend = 0.017; model II). Regarding KEGG pathways, the biosynthesis of ansamycins pathway was identified as a critical pathway that was differentially abundant in healthy controls.

In our study, *Proteobacteria*, *Firmicutes*, *Bacteroidetes*, and *Fusobacterium* were the most dominant phyla in case and control groups. Previous research findings have reported that *Proteobacteria*, *Firmicutes*, *Bacteroidetes*, *Actinobacteria*, and *Fusobacteria* are the most dominant phyla in the gastric environment, which is consistent with the current study [19,22,37,38,39]. We observed that HP was the most dominant species in both the GC cases and controls based on the mean proportion of relative abundance data. This finding is similar to the HP infection status results where 93.4% of controls and 99.6% of GC cases were positive for HP infection based on rapid urease test results. A possible reason for having a higher prevalence of HP infection specifically in healthy controls in our study population needs to be noted. It is generally believed that HP prefers a healthy gastric mucosa, and that as the steps from early stages to late stage of GC progress, HP also gradually fades until it disappears [40]. Furthermore, a multicenter nationwide study conducted using asymptomatic Korean adults between 2015 and 2016 showed that although there was a decreasing trend of HP seropositivity from 1998 to 2015, the HP seroprevalence was still higher than 50% of the population [41]. Thus, it is possible that the HP prevalence might have been remarkably higher both in healthy controls and also the early GC cases in our study. A study conducted using Chinese and Mexican populations revealed that HP was the predominant member of the microbiota in the gastric environment [40]. A study aimed to evaluate the microbial composition of gastric mucosa found that the gastric microbial composition of patients with non-atrophic gastritis, intestinal metaplasia, and GC of the intestinal type harbored *Firmicutes* and *Proteobacteria* phyla, accounting for 70% of in each sample [42]. A similar result was observed in our study, indicating that *Bacteroidetes*, *Firmicutes*, and *Proteobacteria* are dominant phyla in the gastric environment in GC cases and controls. Another study evaluating the gastric microbiota in individuals based on different histopathological stages of GC reported that there were 19 genera with average relative abundances >0.5% across 60 samples at the genus level, where the high-abundance genera were *Helicobacter*, *Flavobacterium*, *Haemophilus*, and *Serratia* [38]. The finding of this study related to the *Helicobacter*, *Haemophilus*, and *Neisseria* genera are consistent with our findings indicating that those three genera are most dominant in both GC cases and control groups. A Korean study observed that the *Epsilonproteobacteria* class corresponding to HP species was predominant, but the abundance of the *Bacilli* class was relatively increased in the GC group, which is consistent with our findings [43]. Thus, our results suggest that, in addition to HP, the endogenous bacterial community might play a major role in the occurrence of GC. An exponential growth of opportunistic bacteria is possible after HP damage to the gastric environment where there is a decrease in acidity of the stomach. Such a disturbance of the microbial community equilibrium can subsequently progress towards GC [44].

A study observed a higher evenness and diversity of the gastric microbiota in the GC group in comparison to chronic gastritis and intestinal metaplasia groups [43]. We observed that the evenness was more or less similar in both GC cases and controls. Interestingly, richness was significantly higher in GC cases in the current study population. A similar finding has been reported in a study comparing cancer tissues with non-cancer tissues, where a high microbial richness was observed in GC tissues [21]. Moreover, we observed a higher Shannon index in the controls compared with the GC group. Another study comparing the chronic gastritis group with the GC group observed a higher Shannon index in the chronic gastritis group compared with the GC group [22]. A study focusing on the relationship between gastric dysbiosis and GC development found an increased richness, although the Shannon index was lower in the GC group compared with the controls, which is similar to our findings [45]. In contrast, a study examining microbiota in gastric mucosa in GC tissues compared with non-cancer tissues revealed a significantly higher Shannon index in the cancer group compared with the healthy controls [21]. Our findings revealed significant microbial community diversity in healthy controls, where the Shannon index of the microbial communities increased significantly. Since gastric carcinoma dysbiosis is generally characterized by reduced microbial diversity, our results demonstrated that the gastric microbiota composition in GC patients was significantly different from healthy controls. It has been further reported that the reduced microbial diversity has now been recognized as a feature of inflammatory-related diseases and several cancers [22]. Thus, our findings for microbial diversity are consistent and validate the results of previously published findings. A study reported that 75.86% of variation in microbiome was captured by the first two principal coordinates in the PCoA beta diversity plot according to the weighted UniFrac phylogenetic distance measure. They observed a significant divergence between non-cancer and cancer samples, since those samples were clustered separately [21]. In a study conducted in Mexico, an ordination analysis of the 44 taxa between non-atrophic gastritis and GC was performed based on the weighted-UniFrac distance measure, and the results showed a significant separation of the microbiota composition between the two groups [42]. These results support our finding that the microbial community structure is different in GC patients compared with healthy controls.

At the species level, *Streptococcus_NCVM* and *C. jejuni* were differentially abundant in the GC cases, while *P. melaninogenica* was differentially abundant in the controls based on the LEfSe analysis. Although the evidence related to the effects of those bacterial species in GC occurrence is limited, a study has reported a remarkable effect of the metabolic products produced by those *Prevotella* species, such as lactic acid, acetic acid, butane diacid, isovaleric acid, and isobutyric acid, to the human gastric cell physiology [46]. Furthermore, *P. melaninogenica* enrichment can create a gastric environment that is more likely to be acidic by lowering the pH than non-atrophic gastritis, where there is a restriction to colonize by other harmful bacterial species [46]. A study evaluated the composition of the microbiota in advanced gastric adenocarcinoma through the shotgun metagenomics approach and reported that the cladogram of the gastric microbiome was phylogenetically associated with GC and superficial gastritis. Family *Porphyromonadaceae*, genus *Porphyromonas*, and genus *Alloprevotella* were enriched in the GC group, whereas genus *Actinomyces* and genus *Atopobium* were enriched in the superficial gastritis group. However, compared with our study, this study did not have similar taxa that were highly enriched, specifically in the GC case group [47]. Based on the cladogram, the *Bacilli* class was phylogenetically related in GC cases in the current study, which is similar to the findings observed by Liu et al. [17]. This finding in our study demonstrates that class *Bacilli* has a potential role in GC carcinogenesis, specifically in Koreans, as another Korean population-based study obtained similar findings [43]. Further, class *Bacilli* plausibly contributes to the production of various metabolic products that can serve as a fuel for the cellular environment to induce angiogenesis [48].

Based on a compositional analysis of microbiome data at the genus level, there was a higher MDI in GC cases than controls that was marginally significant for the total population (*p* = 0.097) and statistically significant for females (*p* = 0.002). Furthermore, the higher MDI showed a significantly increased risk of GC in females (OR: 2.66, 95% CI: 1.19–5.99, *p*-trend = 0.017; model II). A study conducted to observe the association between gastric dysbiosis of the gastric microbiome and GC risk concluded that there is a higher MDI in GC patients than those with chronic gastritis (*p* < 0.0001) [22]. A study conducted to characterize the changes in the microbiome associated with the histopathological stages of gastric tumorigenesis observed significant microbial dysbiosis of the gastric mucosa in GC patients, with a significant overrepresentation of 21 and a reduction of 10 bacterial taxa in GC compared with superficial gastritis (*p* < 0.05) [49].

To derive MDI based on the fold changes in selected genera, *Lactobacillus*, *Diaphorobacter*, *Acinetobacter*, *Atopobium*, *Actinobacillus*, and *Rhizobium* genera were the top six of 13 genera enriched in GC cases in the current study population. Particularly, *Lactobacillus* has a critical role in carcinogenesis because of N-nitroso compounds derived from the metabolism of nitrate/nitrite [28]. In fact, several previous microbiome studies have reported an increase in the abundance of *Lactobacillus* in GC patients [42,43,45]. MDI was significantly positively associated with the risk of GC specifically in females in the current study. As a plausible biological mechanism, it has been reported that the gut microbiome is a principal regulator of circulating estrogen in females [50]. The gut microbiota secretes β-glucuronidase, an enzyme that deconjugates estrogens into their active forms where there is a direct regulation of estrogens by the gut microbiota. Once the dysbiosis of the microbiota has occurred, which is characterized by lower microbial diversity, it can impair the above-mentioned deconjugation process where there is a reduction of the circulating estrogens. The alterations of circulating estrogens may facilitate the development of several pathological conditions, particularly GC, in females [50]. Three possible mechanisms have been proposed for the carcinogenesis resulting from microbial dysbiosis [26].

The first mechanism is related to bacterial-induced chronic inflammation. The inflammatory mediators produced due to chronic inflammation have harmful effects on epithelial and endothelial cells and extracellular matrix compounds. During this inflammatory process, epithelial and immune cells trigger reactive oxygen species (ROSs) and reactive nitrogen species (RNSs) due to the direct influence of TNF-α, IL-6, and TGF-β [51]. Production of ROSs and RNSs occurs via induction of NADPH oxidase and nitric oxide synthase. NADPH oxidase catalyzes the superoxide anion, leading to superoxide dismutase mediated hydrogen peroxide H_2_O_2_ production. Simultaneously, nitric oxide synthase generates nitric oxide (NO), which can be converted into nitrogen dioxide (NO_2_), peroxinitrite, and dinitrogen trioxide (N_2_O_3_), to produce their ROSs and RNSs. Interestingly, increased expression of NADPH oxidase, nitric oxide synthase, and their ROS and RNS species has been identified in the tumor microenvironment [52]. Additionally, cell proliferation, mutagenesis, oncogene activation, and angiogenesis can be facilitated by the inflammatory mediators produced by the above mechanism.

In the second mechanism, NF-κB can be activated, and cellular apoptosis can be inhibited. Activation of the NF-κB pathway that is related to oncogenic cell signaling in epithelial cells has been identified as a critical pathway for TNF- α-induced tumor growth. NF-κB signaling can be categorized into a “classical” pathway and “alternative” pathway. In the classical pathway, IκB kinase β (IKKβ) phosphorylates IκBα, whereas in the alternative pathway, IKKα phosphorylates the p100 precursor of the NF-κB p52 subunit. There is an accumulation of the heterodimeric NF-κB transcription factors in the nucleus as a result of the above signaling events. The classical pathway regulates mainly p50/p65 and p50/c-Rel dimers, and the alternative pathway regulates the p52/relB dimers. Other kinases including the unconventional IKK family members IKKε and TBK1 can also activate the NF-κB pathway. Several signaling pathways converging on the NF-κB regulators provide significant evidence of the ability for cancers to aberrantly stimulate NF-κB [53]. Further, there is an effect on the activation of pro-inflammatory cytokines and oncogenes, including cancer cell proliferation. In the third mechanism, bacterial materials can act as carcinogenic substances that induce the carcinogenesis process [26]. The integration of those mechanisms can potentially stimulate the carcinogenesis process with the involvement of microbial dysbiosis. Particularly, the local concentration of various cytokines, including interleukin-1β (IL-1β), IL-6, and tumor necrosis factor-α (TNF-α) can be increased due to microbial dysbiosis. Endothelial cells can be activated by IL-1β to produce vascular endothelial growth factor (VEGF). VEGF eventually generates an inflammatory microenvironment that is helpful for angiogenesis and tumor progression [54]. Additionally, TNF-α has an ability to produce ROSs that can induce DNA damages. A study focused on the relationship between the tumor-immune environment associated with GC microbiota in patients with GC identified a correlation between regulatory T cells and plasmacytoid dendritic cells in the tumor microenvironment that was further associated with dysbiosis of the gastric microbiota [24].

Regarding the metagenomics functional pathway results, the biosynthesis of ansamycins pathway was highly enriched in controls. It has been reported that ansamycins are groups of antibiotics produced by strains of several *Actinomycetes*. Ansamycins are very potent molecules displaying anticancer, antibacterial, and antiviral activities [55]. The one-carbon pool via the folate pathway was highly enriched in controls compared with GC cases. One-carbon metabolism mediated by folate cofactor supports multiple physiological processes, including the biosynthesis of purines and thymidine, amino acid homeostasis (glycine, serine, and methionine), epigenetic maintenance, and redox defense. While most gut bacteria can synthesize folate, humans require dietary folate intake to maintain physiological processes [56], as it has an essential role in the nucleic acid synthesis. It has been noted that an adequate folate level over the long term may support genome integrity. Based on the evidence of experimental and epidemiological studies, there is a protective effect of folate against colorectal cancer, breast cancer, and pancreatic cancer, while inconsistent results have been reported for GC [57,58,59]. The secondary bile acid biosynthesis pathway was also highly enriched in GC cases compared with controls. Secondary bile acids can induce ROS production, genomic destabilization, apoptosis resistance, and cancer stem cell-like formation. Diverse signals are involved in the carcinogenesis mechanism of bile acids, with a major role of epidermal growth factor receptor and its downstream signaling involving mitogen-activated protein kinase, phosphoinositide 3-kinase/Akt, and nuclear factor kappa-light-chain-enhancer of activated B cells. Bile acids regulate numerous genes including the human leukocyte antigen class I gene, p53, matrix metalloprotease, urokinase plasminogen activator receptor, cyclin D1, cyclooxygenase-2, interleukin-8, and miRNAs of cancer cells [60].

There are several strengths of our study. To the best of our knowledge, this is the first study associated with a methodological approach employing the compositional analysis of microbiome data to derive a MDI for a Korean population (using a novel statistical approach termed “CCREPE”). The main strength of this approach is that it abrogates spurious correlations when determining the significance of a similarity measure. Second, the sample size of the current microbiome-related study is comparatively large relative to previous microbiome studies, with 268 GC cases and 288 healthy subjects improving the power of the statistical analysis to observe the relevant associations between the microbiome and the risk of GC. Third, several potential covariates were considered in a multivariate analysis that are established risk factors for GC development. Those confounding variables were age, smoking, family history of GC, regular exercise, education, occupation, income, and total energy intake throughout the analysis.

There are potential limitations associated with the current study. Generally, selection bias and recall bias need to be raised since this is a hospital-based case-control study. Selection bias might have occurred because healthy subjects were selected from the participants who attended the health screening. They may have had a healthier lifestyle due to health concerns, as compared with individuals who did not participate in screening. Therefore, healthy subjects might be less representative of the general population. Second, the current study was not a follow-up study. Thus, associations between the gastric microbiome and GC risk could have occurred without having a causal relation, as patients with early GC could have changed their microbial profile due to premalignant lesions that had already progressed or because of changes in their dietary habits. However, cases in this study included only patients diagnosed with early GC; thus, the influence of the dietary changes on GC symptoms would have been slight. Third, MDI was not validated, although it was applied in an epidemiological case-control study. Furthermore, since a single sample was measured for the microbial measurements in the current study, the results related to microbial exposure may have had less accuracy compared with the microbiome measurements at multiple time points [61]. In spite of this it is important to note that repeating biopsies with those having a normal gastric histology is associated with ethical issues.

## 4. Materials and Methods

### 4.1. Study Population

Participants were recruited at the National Cancer Center Hospital in Korea between March 2011 and December 2014. Individuals with histologically confirmed early GC within the preceding three months at the Center for Gastric Cancer were included in the case group. Early GC was defined as an invasive carcinoma confined to the mucosa and/or submucosa, regardless of lymph node metastasis status. Patients diagnosed with diabetes mellitus, a history of cancer within the past five years, advanced GC, or severe systemic or mental disease, as well as women who were pregnant or breastfeeding, were excluded. The control group was selected from health-screening examinations at the Center for Cancer Prevention and Detection at the same hospital. Individuals with a history of cancer, diabetes mellitus, gastric ulcers, and HP treatment in the control group were excluded. The final sample of 556 participants was composed of 268 patients and 288 controls (men, 353; women, 203). This study was approved by the Institutional Review Board of the National Cancer Center [IRB Number: NCCNCS-11-438]. Written informed consent was obtained from all participants.

### 4.2. Data Collection

Five gastric mucosa biopsy samples were collected from each study participant following the Sydney system after endoscopy and examination of the stomach. A biopsy sample in the greater curvature, at least 3 cm away from each tumor, was used for the metagenomics analysis. The HP infection status was determined by a rapid urease test, a serological test, and histological evaluation. Regarding the rapid urease test, one biopsy sample was taken from the greater curvature of the corpus. Four biopsy samples were collected from the lesser curvature of the corpus and antrum for histological evaluation. The HP status was determined via Wright–Giemsa staining of the biopsy specimens by a pathologist who specialized in GC. A current infection was defined as at least one positive test result in the rapid urease test or histological evaluation of four biopsy sites [62]. Participants were asked to complete a self-administered questionnaire. Demographic, lifestyle, physical activity, and medical history data were collected from the participants. Total energy intake was obtained from the semi-quantitative food frequency questionnaire (SQFFQ), which has been previously reported as a reliable and valid questionnaire [63].

### 4.3. DNA Extraction

DNA was extracted from the biopsy samples using the MagAttract DNA Blood M48 kit (Qiagen, Hilden, Germany) and BioRobot M48 automatic extraction equipment (Qiagen), according to the manufacturers’ instructions.

### 4.4. Metagenomics 16S rRNA Gene Sequencing

Input gDNA (12.5 ng) was amplified with 16S rRNA gene V3-V4 primers, and a subsequent limited cycle amplification step was performed to add multiplexing indices and Illumina sequencing adapters. The final products were normalized and pooled using PicoGreen, and the library sizes were verified using the LabChip GX HT DNA High Sensitivity Kit (PerkinElmer, Boston, MA, USA). Then, sequencing was performed using the MiSeq platform (Illumina, San Diego, CA, USA). Each sequenced sample was prepared according to the Illumina 16S rRNA gene Metagenomic Sequencing Library protocols. DNA quantification and quality were measured by PicoGreen and Nanodrop analyses, respectively. The 16S rRNA genes were amplified using 16S rRNA gene V3-V4 primers for the 288 control samples and the 268 GC patient samples. The primer sequences were as follows: 16S rRNA gene V3-V4 primer.

16S rRNA gene Amplicon PCR Forward Primer.
5′ TCGTCGGCAGCGTCAGATGTGTATAAGAGACAGCCTACGGGNGGCWGCAG.
16S rRNA gene Amplicon PCR Reverse Primer.
5′ GTCTCGTGGGCTCGGAGATGTGTATAAGAGACAGGACTACHVGGGTATCTAATCC.

The paired-end FASTQ files that had already been demultiplexed were imported to make QIIME2 artifact files. After removing the barcodes/adaptors using Cutadapt, the DADA2 pipeline was applied to remove noisy reads, dereplicate sequences, cluster sequences, and chimeras using QIIME v2.2019.7 [64]. An amplicon sequence variants (ASVs) table was obtained as the end product. Taxonomic abundance was counted with the Ezbio database [65]. Host mitochondrial and chloroplast, archaea, eukaryote, and unassigned reads were filtered before calculating relative abundance. The microbial composition was normalized using the values calculated from the taxonomic abundance count divided by the number of preprocessed reads for each sample to obtain the relative abundance.

### 4.5. Statistical Analysis

#### 4.5.1. Descriptive Statistics

To compare the demographic and lifestyle characteristics between the cases and controls, a chi-square test and Student’s *t*-test were performed for categorical and continuous variables, respectively.

#### 4.5.2. Nonmetric Multidimensional Scaling (NMDS)

NMDS was performed to relate the microbial composition with the metadata of the study population. Ordination distances were calculated based on a Bray–Curtis distance measurement with 10,000 iterations by using metaMDS function of the “vegan” package in R. The Shepard plot was drawn to observe the correlation between observed dissimilarity and the ordination distance. The corresponding stress value was obtained to indicate the goodness of fit for two-dimensional representations of multidimensional space for microbial species. NMDS plots were drawn to observe the difference of microbial composition based on metadata of the study population. The PERMANOVA; adonis function, vegan package in R was used to check the significance of the microbial composition difference based on selected covariates. All statistical analyses were carried out using SAS version 9.4 software (SAS Inc., Cary, NC, USA) and the R platform (version 3.5.1) (R Foundation for Statistical Computing, Vienna, Austria).

#### 4.5.3. Linear Discriminant Analysis of Effect Size (LEfSe) and Cladogram

LEfSe analysis [66] was used to estimate microbiome attributes that differed significantly by cancer status for six taxonomy levels, namely, phylum, class, order, family, genus, and species. LEfSe couples a univariate nonparametric test for statistically significant phenotypic segregation with post hoc prioritization by the size of the effect as determined by LDA. Galaxy implementation of LEfSe [67] with default options was used. Differences were evaluated via a threshold for the logarithmic LDA score for discriminate features for 2.0. An Ezbio feature table was prepared using the Ezbio database to plot the cladogram.

#### 4.5.4. Deriving the MDI

Compositional analysis of microbiome data was performed using CCREPE. This is a novel statistical methodology for co-variation analysis in compositional data [68]. It allows the derivation of accurate significance values for arbitrary association measures (correlation or other similarity scores) when applied to compositional data. It consists of an R package (publicly available through R/Bioconductor–(http://huttenhower.org/ccrepe) and N-dimensional checkerboard score (NC-score), a novel similarity measure specifically designed to detect association patterns in the human microbiome and other microbial communities. This NC-score is an extension of the arbitrary nominal categories of the classical checkerboard score for ecological species co-occurrence. For each pair of microbe m_1_ and m_2_, the NC-score counts the normalized number of co-variation and co-exclusion over all pairs of samples s_1_ and s_2_. The CCREPE method was applied with the relative abundance data set containing 73 genera. CCREPE results were obtained with four matrices (P-values, Z-stat values, NC score, and false discovery rate (FDR) corrected Q values). The sub-correlation matrix of the NC score was extracted according to the two following criteria; FDR corrected Q-values <0.05 and pairs of genera NC score |>0.30|. Finally, 64 genera were selected for further analysis. The fold change of selected genera was calculated by dividing the mean abundance in the cases by that of the controls to identify the genera increased in GC (fold change>1) and decreased in GC (fold change <1), which were presented in a diverging lollipop chart using the R package “ggplot2”. The MDI was calculated as the log of [total abundance in genera increased in GC] over [total abundance in genera decreased in GC].

#### 4.5.5. Association between Gastric Microbiome and GC Risk

The relative abundance of the candidate taxa was categorized into tertiles based on the relative abundance in the control group. Exceptionally, if more than one-third of the subjects had a relative abundance of zero, those bacterial species were categorized into two groups (non-carriers and carriers) based on the median distribution of the controls. Non-carriers were defined as subjects who had a relative abundance of zero. The group with the lowest relative abundance was used as the reference group. The ORs and 95% CIs were estimated using unconditional logistic regression models. The median values of relative abundance in each tertile category were used as continuous variables to test for trends. The OR estimates were calculated for the crude model (model I) and model II. Model II was adjusted for age, smoking, first-degree family history of GC, regular exercise, education, occupation, monthly income, and total energy intake.

#### 4.5.6. Metagenomics Functional Analysis

The gastric microbial functional gene contents were predicted using Phylogenetic Investigation of Communities by Reconstruction of Unobserved States (PICRUSt v2) [69,70]. In brief, the paired-end FASTQ files that were already being demultiplexed were imported to make QIIME2 artifact files. After removing the barcodes/adaptors using Cutadapt, the DADA2 pipeline was applied to remove noisy reads, dereplicate sequences, cluster sequences, and chimeras using QIIME v2.2019.7 [64]. An amplicon sequence variant (ASV) table was obtained as the end product. It was normalized, and the metagenome functional predictions were predicted via PICRUSt v2 [71].

## 5. Conclusions

The current results show that specific bacterial pathogens may play a role in GC risk, whereas other bacterial types may be associated with reduced risk. Moreover, a significantly higher MDI was observed in GC cases compared with controls, and MDI was significantly positively associated with GC risk in the female population. Microbial composition was significantly different based on HP infection status. Particularly, microbial metabolic pathways in the gastric microbiota were associated with GC. Specifically, the biosynthesis of ansamycins pathway was highly enriched in the GC cases. Evaluating the gastric microbial composition associated with GC will be beneficial to developing novel preventive guidelines to prevent GC risk based on the individual microbiome profiles of Koreans in the future.

## Figures and Tables

**Table 1:** Comparison of diversity measures between gastric cancer (GC) cases and controls.

Alpha Diversity Indices	All (*n* = 556)		*p*-Value
	Controls (*n* = 288)	Cases (*n* = 268)	
Shannon index	2.06 ± 2.59	1.66 ± 1.06	0.030
Richness	32.25 ± 16.93	35.89 ± 16.00	0.009
Evenness	0.14 ± 0.06	0.15 ± 0.03	0.440
Pilou evenness	0.58 ± 0.61	0.46 ± 0.37	0.004
	Male (*n* = 353)		
	Controls (*n* = 181)	Cases (*n* = 172)	
Shannon index	1.93 ± 2.37	1.79 ± 1.77	0.519
Richness	31.55 ± 16.44	38.26 ± 16.25	<0.001
Evenness	0.14 ± 0.07	0.15 ± 0.03	0.560
Pilou evenness	0.55 ± 0.55	0.48 ± 0.40	0.190
	Female (*n* = 203)		
	Controls (*n* = 107)	Cases (*n* = 96)	
Shannon index	2.27 ± 2.93	1.43 ± 1.21	0.007
Richness	33.44 ± 17.76	31.66 ± 14.72	0.440
Evenness	0.14 ± 0.03	0.14 ± 0.03	0.578
Pilou evenness	0.63 ± 0.69	0.42 ± 0.28	0.004

**Table 2:** Association between relative abundance of bacterial species and gastric cancer (GC) risk.

Candidate Species	No. of Controls (%)	No. of Cases (%)	Model I OR (95% CI)	Model II OR (95% CI)
*Campylobacter jejuni*				
0 (Non-carriers)	288(100.0)	264(98.5)	1.00	1.00
>0 (Carriers)	0(0.0)	4(1.5)	>999.99(<0.001–>999.99)	>999.99(<0.001–>999.99)
*Streptococcus_CP003667*				
0 (Non-carriers)	187(64.9)	203(75.8)	1.00	1.00
> 0 (Carriers)	101(35.1)	65(24.3)	0.59(0.41–0.86)	0.58(0.38–0.88)
*Gemella taiwanensis*				
0 (Non-carriers)	155(53.8)	118(44.0)	1.00	1.00
>0 (Carriers)	133(46.2)	150(55.9)	1.48(1.06–2.07)	1.10(0.75–1.62)
*Streptococcus_NCVM*				
<0.000458	95(33.0)	62(23.1)	1.00	1.00
0.000458–0.00204	96(33.3)	92(34.3)	1.47(0.96–2.26)	1.26(0.78–2.03)
≥0.00204	97(33.7)	114(42.5)	1.80(1.18–2.74)	1.37(0.85–2.21)
*p*-trend			0.022	0.308
*Prevotella intermedia*				
0 (Non-carriers)	133(46.2)	144(53.7)	1.00	1.00
>0 (Carriers)	155(53.8)	124(46.3)	0.74(0.53–1.03)	0.69(0.47–1.00)
*Prevotella melaninogenica*				
<0.000356	95(33.0)	113(42.2)	1.00	1.00
0.000356–0.00178	96(33.3)	77(28.7)	0.67(0.45–1.01)	0.68(0.43–1.07)
≥0.00178	97(33.7)	78(29.1)	0.68(0.45–1.01)	0.91(0.38–0.96)
*p*-trend			0.141	0.071
*Prevotella nigrescens*				
0 (Non-carriers)	157(54.50	179(66.8)	1.00	1.00
>0 (Carriers)	1319(45.5)	89(33.2)	0.60(0.42–0.84)	0.64(0.43–0.94)
*Streptococcus vestibularis*				
0 (Non-carriers)	153(53.1)	81(30.2)	1.00	1.00
>0 (Carriers)	135(46.9)	187(69.8)	2.62(1.85–3.71)	2.41(1.63–3.56)

Model I: Crude; Model II: Adjusted for age, family history of GC, regular exercise, education, occupation, income, total energy intake. OR: odds ratio, CI: confidence interval.

**Table 3:** Comparison of the Microbial dysbiosis index (MDI) between cases and controls.

Microbial dysbiosis index (MDI)	Cases	Controls	*p*-Value
Total (*N*)	268	288	
MDI	3.77 ± 1.94	3.45 ± 2.59	0.097
Male (*N*)	172	181	
MDI	3.52 ± 2.04	3.58 ± 2.43	0.773
Female (*N*)	96	107	
MDI	4.23 ± 1.65	3.22 ± 2.84	0.002

MDI: Microbial dysbiosis index.

**Table 4:** Association between the microbial dysbiosis index (MDI) and gastric cancer (GC) risk.

Microbial dysbiosis index (MDI)	No. of Controls (%)	No. of Cases (%)	Model I	Model II
Total				
T1(<3.18)	96(33.3)	91(33.9)	1.00	1.00
T2(3.18–4.52)	97(33.7)	75(27.9)	0.82(0.54–1.24)	0.97(0.60–1.57)
T3(≥4.52)	95(33.0)	102(38.1)	1.13(0.76–1.69)	1.37(0.86–2.17)
*p* for trend			0.561	0.179
Male				
T1(<3.25)	60(33.2)	74(43.0)	1.00	1.00
T2(3.25–4.48)	60(33.2)	42(24.4)	0.57(0.34–0.96)	0.80(0.43–1.52)
T3(≥4.48)	61(33.7)	56(32.6)	0.74(0.45–1.22)	1.15(0.63–2.11)
*p* for trend			0.225	0.657
Female				
T1(<3.04)	36(33.6)	18(18.8)	1.00	1.00
T2(3.04–4.52)	36(33.6)	31(32.3)	1.72(0.82–3.62)	1.69(0.71–4.02)
T3(≥4.52)	35(32.7)	47(48.9)	2.69(1.31–5.49)	2.66(1.19–5.99)
*p* for trend			0.006	0.017

Model I: Crude; Model II: Adjusted for age, family history of GC, regular exercise, education, occupation, income, total energy intake.

## Supplementary Materials

The following are available online at https://www.mdpi.com/2072-6694/12/9/2619/s1, Table S1. General characteristics of the study population. Table S2: Fold change values for the candidate genera that were increased in GC. Table S3: Fold change values for the candidate genera that were decreased in GC. Table S4: Stratified analysis of the association between microbial dysbiosis index (MDI) and GC risk based on lifestyle factors. Table S5: Mean relative abundance of Kyoto Encyclopedia of Genes and Genomes (KEGG) pathways identified by LEfSe and KEGG orthologies (KOs). Figure S1: Bar plot of the taxonomic profiles between GC cases and controls at the species level. Figure S2: Principal coordinate analysis (PCoA) plot of the Bray-Curtis distance. The red dots indicate the cases, while the blue triangles indicate the controls. The red and blue ellipses represent where 95% of the data belong to controls and cases, respectively, at 5% significance.
