# Supplementary material for: Alterations in Gastric Microbial Communities Are Associated with Risk of Gastric Cancer in a Korean Population: A Case-Control Study
Source: Cancers (Basel). 2020 Sep 14;12(9):2619. doi: 10.3390/cancers12092619 (PMC7563352; doi:10.3390/cancers12092619)
Supplement: Supplementary file 1 [file cancers-12-02619-s001.pdf]

Supplementary file:

Table S1. General characteristics of the study population

	All (n=556)			Male (n=353)			Female (n=203)		
Variable	Control (n=288)	Cases (n=268)	p-value	Control (n=181)	Cases (n=172)	p-value	Control (n=107)	Cases (n=96)	p-value
Age (y)	51.53±7.21	53.68±9.60	0.003	52.07±6.46	54.69±8.86	0.002	50.62±8.29	51.86±10.59	0.355
<50	114(39.58)	93(34.70)	0.234	65(35.91)	52(30.23)	0.257	49(45.79)	41(42.71)	0.657
≥50	174(60.42)	175(65.30)		116(64.09)	120(69.77)		58(54.21)	55(57.29)	
Sex [n (%)]			0.745						
Male	181(62.85)	172(64.18)							
Female	107(37.15)	96(35.82)							
Body Mass index (kg/m²) [n (%)]	23.99±3.11	23.91±3.02	0.747	24.48±3.04	24.30±2.85	0.573	23.18±3.07	23.21±3.20	0.939
<23	113(39.24)	107(39.93)	0.863	58(32.04)	58(33.72)	0.931	55(51.40)	49(51.04)	0.802
23-25	81(28.13)	70(26.12)		51(28.18)	46(26.74)		30(28.04)	24(25.00)	
≥25	94(32.64)	91(33.96)		72(39.78)	68(39.53)		22(20.56)	23(23.96)	
Missing									
Smoking status [n (%)]			0.006			0.006			0.243
Current smoker	51(17.71)	78(29.10)		50(27.62)	75(43.60)		1(0.93)	3(3.13)	
Ex-smoker	98(34.03)	80(29.85)		95(52.49)	74(43.02)		3(2.80)	6(6.25)	
Nonsmoker	139(48.26)	109(40.67)		36(19.89)	23(13.37)		103(96.26)	86(89.58)	
Missing	0(0.00)	1(0.37)					0(0.00)	1(1.04)	
Alcohol consumption [n (%)]			0.559			0.618			0.860
Current drinker	184(63.89)	163(60.82)		137(75.69)	123(71.51)		47(43.93)	40(41.67)	
Ex-drinker	21(7.29)	26(9.70)		17(9.39)	21(12.21)		4(3.74)	5(5.21)	
Nondrinker	83(28.82)	78(29.10)		27(14.92)	28(16.28)		56(52.34)	50(52.08)	
Missing	0(0.00)	1(0.37)					0(0.00)	1(1.04)	
Family history of GC			0.003			0.015			0.112
Yes	34(11.81)	56(20.90)		25(13.81)	41(23.84)		9(8.41)	15(15.63)	
No	254(88.19)	211(78.73)		156(86.19)	130(75.58)		98(91.59)	81(84.38)	
Missing	0(0.0)	1(0.37)							

Regular exercise [n (%)]			<0.001			0.079			<0.001
Yes	150(52.08)	95(35.45)		89(49.17)	69(40.12)		61(57.01)	26(27.08)	
No	137(47.57)	173(64.55)		91(50.28)	103(59.88)		46(42.99)	70(72.92)	
Missing	1(0.35)	0(0.00)		1(0.55)	0(0.00)				
Educational level [n (%)]			<0.001			<0.001			0.001
Middle school	42(14.58)	92(34.33)		25(13.81)	58(33.72)		17(15.89)	34(35.42)	
High school	86(29.86)	116(43.28)		43(23.76)	77(44.77)		43(40.19)	39(40.63)	
College or more	148(51.39)	58(21.64)		103(56.91)	36(20.93)		45(42.06)	22(22.92)	
Missing	12(4.17)	2(0.75)		10(5.52)	1(0.58)		2(1.87)	1(1.04)	
Occupation [n (%)]			0.037			0.004			0.017
Group1:Professionals, administrative management	60(20.83)	44(16.42)		45(24.86)	37(21.51)		15(14.02)	7(7.29)	
Group2:Office, Sales and service positions	98(34.03)	72(26.87)		74(40.88)	46(26.74)		24(22.43)	26(27.08)	
Group3:Agriculture, laborer	47(16.32)	65(24.25)		43(23.76)	51(29.65)		4(3.74)	14(14.58)	
Group4:Unemployment and others	83(28.82)	85(31.72)		19(10.50)	37(21.51)		64(59.81)	48(50.00)	
Missing	0(0.00)	2(0.75)		0(0.00)	1(0.58)		0(0.00)	1(1.04)	
Marital status [n (%)]			0.319			0.249			0.864
Married	245(85.07)	234(87.31)		157(86.74)	155(90.12)		88(82.24)	79(82.29)	
Others (single, divorced, separated, widowed, cohabitating)	43(14.93)	32(11.94)		24(13.26)	16(9.30)		19(17.76)	16(16.67)	
Missing	0(0.00)	2(0.75)		0(0.00)	1(0.58)		0(0.00)	1(1.04)	
Monthly income [n (%)]*			<0.001			<0.001			0.084
<200	46(15.97)	79(29.48)		21(11.60)	49(28.49)		25(23.36)	30(31.25)	
200 -400	114(39.58)	101(37.69)		80(44.20)	70(40.70)		34(31.78)	31(32.29)	
≥400	110(38.19)	59(22.01)		64(35.36)	34(19.77)		46(42.99)	25(26.04)	
Missing	18(6.25)	29(10.82)		16(8.84)	19(11.05)		2(1.87)	10(10.42)	
HP infection			<0.001			0.008			0.004
Positive	269(93.40)	267(99.63)		171(94.48)	171(99.42)		98(91.59)	96(100.00)	
Negative	19(6.60)	1(0.37)		10(5.52)	1(0.58)		9(8.41)	0(0.00)	
Missing									
Supplements use [n(%)]			0.416			0.342			0.892
Yes	159(55.21)	157(58.58)		92(50.83)	96(55.81)		67(62.62)	61(63.54)	

No	127(44.10)	109(40.67)		87(48.07)	74(43.02)		40(37.38)	35(36.46)	
Missing	2(0.69)	2(0.75)		2(1.10)	2(1.16)				
Lauren's classification			NA			NA			NA
Intestinal	NA	105(39.18)		NA	89(51.74)		NA	16(16.67)	
Diffuse	NA	109(40.67)		NA	51(29.65)		NA	58(60.42)	
Mixed	NA	36(13.43)		NA	21(12.21)		NA	15(15.63)	
Missing	NA	18(6.72)		NA	11(6.40)		NA	7(7.29)	
Total Energy intake (Kcal/day)	1766.35±554.67	1934.24±624.91	<0.001	1839.30±542.53	2057.70±643.65	0.001	1642.95±555.62	1713.03±524.18	0.358

Values are expressed as mean ±standard deviation (SD) or n (%). *Unit is 10,000 Won in Korean currency. Exchange rate 1 US$=1122 Korean Won (February 2019). **p values were calculated by using Student's t-test for continuous variables and chi-square test for categorical variables.

Table S2. Fold change values for the candidate genera that were increased in GC

Genera	Cases	Controls	Fold change
Atopobium	0.000133	0.00007	1.90
Anaerobacillus	0.000095	0.000086	1.10
Lactobacillus	0.00333	0.00007	47.57
Bradyrhizobium	0.000074	0.000071	1.04
Ochrobactrum	0.000069	0.000061	1.13
Rhizobium	0.000013	0.00001	1.30
Aquabacterium	0.000038	0.000032	1.19
Diaphorobacter	0.000027	0.000011	2.45
Variovorax	0.000813	0.000788	1.03
Helicobacter	0.916	0.879	1.04
Actinobacillus	0.000842	0.000642	1.31
Acinetobacter	0.000322	0.000165	1.95
GU410548_g	0.00052	0.00052	1.00

Fold change = [Mean relative abundance of a genus in cases] / [Mean relative abundance of a genus in controls]

Table S3. Fold change values for the candidate genera that were decreased in GC

Genera	Cases	Controls	Fold change
Actinomyces	0.000216	0.000367	0.59
Corynebacterium	0.000054	0.000077	0.70
Rothia	0.00056	0.000951	0.59
Cutibacterium	0.000018	0.000074	0.24
Bacteroides	0.000095	0.0001	0.95
Paludibacter	0.000015	0.000051	0.29
Porphyromonas	0.0026	0.00542	0.48
Tannerella	0.000078	0.000123	0.63
Alloprevotella	0.0042	0.00778	0.54
Prevotella	0.0123	0.0266	0.46
Bergeyella	0.000264	0.00032	0.83
Capnocytophaga	0.0005	0.00124	0.40
Gemella	0.000938	0.00114	0.82
Granulicatella	0.000295	0.000354	0.83
Streptococcus	0.01	0.0101	0.99
PAC001141	0.000029	0.00005	0.58
Butyrivibrio	3.33E-07	1.08E-06	0.31
Catonella	0.000091	0.000219	0.42
Lachnoanaerobaculum	0.000019	0.000067	0.28
Moryella	0.000043	0.000095	0.45
Oribacterium	0.000102	0.000225	0.45
Aminicella	7.57E-06	0.000039	0.19
Eubacterium_g10	0.000048	0.000079	0.61
Filifactor	0.000063	0.000232	0.27
Peptoanaerobacter	0.000027	0.000076	0.36
Peptostreptococcus	0.000087	0.000092	0.95
PAC000661	0.000094	0.000279	0.34
Bulleidia	0.000083	0.000101	0.82
Selenomonas	0.00029	0.00052	0.56
Dialister	0.000064	0.000193	0.33
Megasphaera	0.000433	0.000458	0.95
Veillonella	0.00459	0.00756	0.61
Parvimonas	0.000038	0.000048	0.79
Fusobacterium	0.00252	0.00514	0.49
Leptotrichia	0.00118	0.00227	0.52
JN713562_g	0.000031	0.000045	0.69
Brevundimonas	0.000019	0.000021	0.90
Bosea	0.000227	0.000254	0.89
Delftia	0.000616	0.000629	0.98

Lautropia	0.00006	0.000256	0.23
Ralstonia	0.000757	0.000765	0.99
Kingella	0.00005	0.000059	0.85
Neisseria	0.0119	0.019	0.63
Campylobacter	0.000895	0.00103	0.87
Cardiobacterium	0.000021	0.00003	0.70
Aggregatibacter	0.000588	0.000694	0.85
Haemophilus	0.0152	0.0181	0.84
PAC000677_g	0.000091	0.000162	0.56
Saccharimonas	0.000338	0.000614	0.55
Treponema	0.000253	0.00053	0.48
Fretibacterium	0.000025	0.000059	0.42

Fold change = [Mean relative abundance of a genus in cases] / [Mean relative abundance of a genus in controls]

Table S4. Stratified analysis of the association between microbial dysbiosis index (MDI) and GC risk based on lifestyle factors

Microbial dysbiosis index (MDI)	No. of controls (%)	No. of cases (%)	Model I	Model II
Age				
Young [<50Y]				
T1(<3.06)	37(32.5)	26(28.0)	1.00	1.00
T2(3.06-4.47)	38(33.3)	24(25.8)	0.90(0.44-1.84)	1.03(0.46-2.29)
T3(≥4.52)	39(34.2)	43(46.2)	1.57(0.81-3.05)	1.65(0.77-3.57)
p for trend			0.174	0.192
Old [>50Y]				
T1(<3.07)	57(32.8)	57(32.6)	1.00	1.00
T2(3.07-4.41)	58(33.3)	55(31.4)	0.95(0.56-1.60)	0.97(0.52-1.79)
T3(≥4.41)	59(33.9)	63(36.0)	1.07(0.64-1.78)	1.31(0.71-2.43)
p for trend			0.789	0.374
Body mass index (BMI)				
<23				
T1(<3.40)	38(33.7)	36(33.6)	1.00	1.00
T2(3.40-4.61)	37(32.7)	26(24.3)	0.74(0.38-1.46)	1.14(0.50-2.58)
T3(≥4.61)	38(33.6)	45(42.1)	1.25(0.67-2.34)	1.96(0.90-4.27)
p for trend			0.518	0.091
23-25				
T1(<2.95)	27(33.3)	16(22.8)	1.00	1.00
T2(2.95-4.18)	27(33.3)	24(34.3)	1.50(0.66-3.43)	1.16(0.42-3.17)
T3(≥4.18)	28(33.3)	30(42.9)	1.88(0.84-4.21)	1.58(0.58-4.27)
p for trend			0.128	0.362
>25				
T1(<3.04)	32(34.0)	34(37.4)	1.00	1.00
T2(3.04-4.58)	31(33.0)	32(35.2)	0.97(0.49-1.94)	1.26(0.54-2.89)
T3(≥4.58)	31(33.0)	25(27.5)	0.76(0.37-1.55)	0.82(0.36-1.88)
p for trend			0.466	0.676
Smoking				
Ever smoking				
T1(<3.27)	51(34.2)	67(42.4)	1.00	1.00
T2(3.27-4.48)	48(32.2)	38(24.1)	0.60(0.34-1.06)	0.81(0.41-1.60)
T3(≥4.48)	50(33.6)	53(33.5)	0.81(0.47-1.37)	1.18(0.61-2.28)
p for trend			0.448	0.597
Never smoking				
T1(<2.99)	47(33.8)	24(22.0)	1.00	1.00
T2(2.99-4.52)	46(33.1)	35(32.1)	1.49(0.77-2.88)	1.63(0.75-3.56)
T3(≥4.52)	46(33.1)	50(45.9)	2.13(1.13-4.01)	2.37(1.14-4.92)

p for trend			0.019	0.019
Drinking				
Ever drinking				
T1(<3.18)	68(33.2)	70(37.0)	1.00	1.00
T2(3.18-4.52)	69(33.7)	49(25.9)	0.69(0.42-1.13)	0.85(0.48-1.53)
T3(≥4.52)	68(33.2)	70(37.0)	1.00(0.62-1.60)	1.14(0.66-1.98)
p for trend			0.961	0.630
Never drinking				
T1(<3.17)	27(32.5)	21(26.9)	1.00	1.00
T2(3.17-4.52)	28(33.7)	25(32.1)	1.15(0.52-2.52)	0.93(0.37-2.34)
T3(≥4.52)	28(33.7)	32(41.0)	1.47(0.68-3.15)	1.82(0.74-4.47)
p for trend			0.317	0.175
Family history of GC				
Yes				
T1(<2.81)	11(32.4)	17(30.4)	1.00	1.00
T2(2.81-4.53)	12(35.3)	21(37.5)	1.13(0.40-3.19)	0.92(0.24-3.62)
T3(≥4.53)	11(32.4)	18(32.1)	1.06(0.36-3.08)	1.17(0.24-5.57)
p for trend			0.874	0.922
No				
T1(<3.28)	86(33.8)	71(33.6)	1.00	1.00
T2(3.28-4.52)	83(32.6)	56(26.5)	0.82(0.52-1.29)	1.05(0.62-1.77)
T3(≥4.52)	85(33.5)	84(39.8)	1.19(0.77-1.85)	1.41(0.86-2.32)
p for trend			0.423	0.169
Regular exercise				
Yes				
T1(<3.00)	50(33.3)	37(38.9)	1.00	1.00
T2(3.00-4.51)	50(33.3)	28(29.5)	0.76(0.40-1.42)	0.89(0.42-1.89)
T3(≥4.51)	50(33.3)	30(31.6)	0.81(0.44-1.51)	1.45(0.69-3.06)
p for trend			0.468	0.361
No				
T1(<3.24)	46(33.6)	50(28.9)	1.00	1.00
T2(3.24-4.55)	45(32.9)	50(28.9)	1.02(0.58-1.80)	1.50(0.76-2.96)
T3(≥4.55)	46(33.6)	73(42.2)	1.46(0.85-2.52)	1.65(0.88-3.11)
p for trend			0.154	0.137
IPAQ categorical scale				
Low				
T1(<3.17)	33(33.0)	31(26.7)	1.00	1.00
T2(3.17-4.49)	33(33.0)	33(28.7)	1.07(0.54-2.12)	1.08(0.47-2.48)
T3(≥4.49)	34(34.0)	51(44.4)	1.60(0.83-3.07)	1.75(0.81-3.81)
p for trend			0.154	0.145
Moderate				
T1(<2.95)	35(32.4)	28(33.3)	1.00	1.00
T2(2.95-4.29)	36(33.3)	21(25.0)	0.73(0.35-1.52)	0.76(0.33-1.75)

T3(≥4.29)	37(34.3)	35(41.7)	1.18(0.60-2.33)	1.59(0.72-3.48)
p for trend			0.650	0.263
High				
T1(<3.52)	27(33.8)	33(47.8)	1.00	1.00
T2(3.52-4.77)	27(33.8)	19(27.5)	0.58(0.27-1.25)	1.00(0.34-2.94)
T3(≥4.77)	26(32.5)	17(24.6)	0.54(0.24-1.19)	0.52(0.18--1.52)
p for trend			0.104	0.243
Education				
Middle school				
T1(<2.24)	14(33.3)	22(23.9)	1.00	1.00
T2(2.24-4.56)	14(33.3)	40(43.5)	1.82(0.74-4.50)	1.78(0.63-5.03)
T3(≥4.56)	14(33.3)	30(32.6)	1.36(0.54-3.43)	1.38(0.47-4.09)
p for trend			0.596	0.577
High school				
T1(<3.18)	28(32.6)	39(33.6)	1.00	1.00
T2(3.18-4.51)	30(34.9)	34(29.3)	0.81(0.41-1.62)	1.10(0.50-2.44)
T3(≥4.51)	28(32.6)	43(37.1)	1.10(0.56-2.18)	1.38(0.63-3.03)
p for trend			0.787	0.412
College or more				
T1(<3.39)	50(33.8)	16(27.6)	1.00	1.00
T2(3.39-4.51)	48(32.4)	13(22.4)	0.85(0.37-1.95)	0.86(0.36-2.05)
T3(≥4.51)	50(33.8)	29(50.0)	1.81(0.88-3.74)	1.91(0.89-4.11)
p for trend			0.081	0.075
Occupation*				
Group 1				
T1(<3.42)	20(33.3)	19(43.2)	1.00	1.00
T2(3.42-4.85)	20(33.3)	13(29.6)	0.68(0.27-1.75)	1.01(0.31-3.29)
T3(≥4.85)	20(33.3)	12(27.3)	0.63(0.24-1.64)	0.89(0.27-2.98)
p for trend			0.334	0.853
Group 2				
T1(<2.93)	34(34.7)	20(27.8)	1.00	1.00
T2(2.93-4.30)	31(31.6)	18(25.0)	0.98(0.44-2.20)	1.25(0.52-3.01)
T3(≥4.30)	33(33.7)	34(47.2)	1.75(0.84-3.64)	1.89(0.83-4.33)
p for trend			0.114	0.124
Group 3				
T1(<3.37)	16(34.0)	28(43.1)	1.00	1.00
T2(3.37-4.92)	16(34.0)	16(24.6)	0.57(0.23-1.44)	0.73(0.23-2.33)
T3(≥4.92)	15(31.9)	21(32.3)	0.80(0.32-1.98)	1.19(0.36-4.04)
p for trend			0.588	0.795
Group 4				
T1(<3.28)	28(33.7)	25(29.4)	1.00	1.00
T2(3.28-4.55)	27(32.5)	27(31.8)	1.12(0.53-2.39)	1.93(0.72-5.16)
T3(≥4.55)	28(33.7)	33(38.8)	1.32(0.63-2.76)	2.51(0.98-6.44)

p for trend			0.464	0.056
Marital status				
Married				
T1(<3.24)	82(33.5)	79(33.8)	1.00	1.00
T2(3.24-4.49)	81(33.1)	63(26.9)	0.81(0.51-1.27)	0.91(0.54-1.53)
T3(≥4.49)	82(33.5)	92(39.3)	1.17(0.75-1.79)	1.38(0.84-2.26)
p for trend			0.517	0.211
Others				
T1(<2.78)	14(32.6)	8(25.0)	1.00	1.00
T2(2.78-4.92)	14(32.6)	16(50.0)	2.00(0.65-6.17)	4.23(0.83-21.56)
T3(≥4.92)	15(34.9)	8(25.0)	0.93(0.28-3.17)	2.12(0.34-13.08)
p for trend			0.751	0.497
Monthly income**				
<200				
T1(<3.42)	16(34.8)	34(43.0)	1.00	1.00
T2(3.42-4.92)	15(32.6)	24(30.4)	0.75(0.31-1.81)	0.57(0.19-1.66)
T3(≥4.92)	15(32.6)	21(26.6)	0.66(0.27-1.60)	0.48(0.16-1.45)
p for trend			0.347	0.181
200-400				
T1(<3.16)	38(33.3)	28(27.7)	1.00	1.00
T2(3.16-4.48)	38(33.3)	30(29.7)	1.07(0.54-2.12)	1.64(0.73-3.67)
T3(≥4.48)	38(33.3)	43(42.6)	1.54(0.79-2.96)	2.09(0.97-4.53)
p for trend			0.192	0.062
>400				
T1(<3.24)	37(33.6)	16(27.1)	1.00	1.00
T2(3.24-4.49)	36(32.7)	15(25.4)	0.96(0.42-2.23)	0.86(0.34-2.17)
T3(≥4.49)	37(33.6)	28(47.5)	1.75(0.81-3.76)	1.92(0.81-4.58)
p for trend			0.156	0.154
HP relative abundance				
Low				
T1(<3.17)	49(32.7)	48(37.5)	1.00	1.00
T2(3.17-4.49)	51(34.0)	46(35.9)	0.92(0.52-1.62)	0.95(0.48-1.89)
T3(≥4.49)	50(33.3)	34(26.6)	0.69(0.38-1.25)	0.95(0.47-1.94)
p for trend			0.314	0.884
High				
T1(<4.59)	47(34.1)	42(30.0)	1.00	1.00
T2(4.59-5.47)	45(32.6)	51(36.4)	1.27(0.71-2.26)	1.35(0.71-2.59)
T3(≥5.47)	46(33.3)	47(33.6)	1.14(0.64-2.05)	1.04(0.54-1.99)
p for trend			0.671	0.945
Total energy intake				
Low				
T1(<3.04)	53(36.8)	36(31.0)	1.00	1.00
T2(3.04-4.48)	47(32.6)	37(31.9)	1.16(0.63-2.12)	1.49(0.73-3.03)

T3(≥4.48)	44(30.6)	43(37.1)	1.44(0.79-2.61)	1.86(0.93-3.71)
p for trend			0.234	0.078
High				
T1(<3.34)	49(34.0)	58(38.2)	1.00	1.00
T2(3.34-4.71)	47(32.6)	38(25.0)	0.68(0.38-1.21)	0.85(0.44-1.66)
T3(≥4.71)	48(33.3)	56(36.8)	0.98(0.57-1.69)	1.13(0.59-2.15)
p for trend			0.943	0.728

Model I: Crude model

Model II: Adjusted for Age, sex, family history of GC, smoking status, regular exercise, education, occupation, income and total energy intake

*Group1: Professionals, administrative management; Group2: Office, Sales and service positions; Group3: Agriculture, laborer; Group4: Unemployment and others

**Unit is 10,000 Won in Korean currency

Table S5. Mean relative abundance of Kyoto Encyclopedia of Genes and Genomes (KEGG) pathways identified by LEfSe and KEGG orthologies (KOs)

KEGG pathway	Pathway name	Cases (268)	Controls (288)	p-value*	KEGG Orthology	KO Name	Cases (268)	Controls (288)	p-value*
ko01051	Biosynthesis of ansamycins	0.0292±0.0013	0.0297±0.0014	<0.001	K00615	Transketolase	0.0011±0.000054	0.0018±0.00005	0.0041
ko00670	One carbon pool by folate	0.0150±0.0011	0.0156±0.0016	<0.001	K01492	Phosphoribosylglycinamide	8.07E-7±2.05E-6	7.18E-7±2.18E-6	<0.001
					K13938	Dihydromonapterin reductase	7.51E-8±2.53E-7	6.14E-8±3.54E-7	<0.001
					K13990	Glutamate formiminotransferase	0.000019±0.00004	0.000033±0.000073	0.022
ko00550	Peptidoglycan biosynthesis	0.0201±0.0005	0.0203±0.0006	<0.001	K00687	Penicillin-binding protein 2B	0.000013±0.000027	0.000013±0.000046	0.011
					K00790	UDP-N-acetylglucosamine 1-carboxyvinyltransferase	0.0011±0.000034	0.0011±0.000048	0.039
					K00887	Undecaprenol kinase	0.000017±0.000044	0.000015±0.000052	0.008
					K03693	Penicillin-binding protein 1B	0.000017±0.000038	0.000015±0.000052	0.011
					K05362	UDP-N-acetylmuramoyl-L-alanyl-D-glutamate-L-lysine ligase	0.000016±0.000042	0.000013±0.000046	0.009
					K05363	Serine/alanine adding enzyme	1.74E-6±3.77E-6	1.71E-6±4.42E-6	<0.001
					K05364	Penicillin-binding protein A	2.36E-6±5.14E-6	2.95E-6±7.28E-6	<0.001
					K05366	Penicillin-binding protein 1A	0.0011±0.000047	0.0011±0.000070	0.009
					K05515	Penicillin-binding protein 2	0.0010±0.000098	0.0010±0.000087	<0.001
					K07009	Lipid II isoglutaminyl synthase	0.000023±0.000074	0.000018±0.000062	0.002
					K07260	Zinc D-Ala-D-Ala carboxypeptidase	0.000018±0.000032	0.000019±0.000063	0.009
					K08724	Penicillin-binding protein 2B	5.48E-6±0.000057	2.66E-6±0.000017	0.045
					K12554	Alanine adding enzyme	1.80E-6±3.87E-6	2.26E-6±0.000011	<0.001
					K12555	Penicillin-binding protein 2A	0.000018±0.000063	0.000014±0.000051	0.009
					K12556	Penicillin-binding protein 2X	0.000013±0.000027	0.000013±0.000046	0.011
					K18149	Penicillin-binding protein	6.83E-7±1.65E-6	2.32E-6±0.00003	0.038
					K18770	Penicillin-binding protein 4	1.55E-7±2.78E-7	1.25E-7±2.65E-7	0.033

ko00540	Lipopolysaccharide biosynthesis	0.0277±0.0031	0.0278±0.0033	0.028	K00677	UDP-N-acetylglucosamine acyltransferase	0.0010±0.000098	0.0011±0.000089	<0.001
					K00713	Alpha-1,2-glucosyltransferase	4.68E-8±1.44E-7	4.43E-8±2.78E-7	<0.001
					K02517	Lauroyltransferase/acyltransferase	0.0011±0.000092	0.0011±0.000078	0.002
					K02848	Heptose I phosphotransferase	6.85E-8±2.51E-7	5.31E-8±2.88E-7	<0.001
					K03275	Alpha-1,3-glucosyltransferase	4.82E-8±1.44E-7	4.87E-8±2.79E-7	<0.001
					K03276	Alpha-1,2-Glucosyl/galactosyltransferase	4.97E-8±1.47E-7	4.89E-8±2.81E-7	<0.001
					K03278	Alpha-1,3-D-galactosyltransferase	4.69E-8±1.44E-7	4.77E-8±2.78E-7	<0.001
					K03279	Alpha-1,2-glucosyltransferase	9.25E-6±0.00002	0.000013±0.000041	<0.001
					K03760	Lipid A ethanolaminephosphotransferase	0.000022±0.00012	6.26E-6±0.000014	0.012
					K07264	4-amino-4-deoxy-L-arabinose transferase	7.94E-8±3.84E-7	6.15E-8±3.48E-7	<0.001
					K09953	Lipid A 3-O-deacylase	4.69E-8±1.44E-7	4.77E-8±2.78E-7	<0.001
					K12973	Lipid IVA palmitoyltransferase	5.68E-8±1.50E-7	6.09E-8±3.47E-7	<0.001
					K12974	KDO2-lipid IV(A) palmitoleoyltransferase	5.41E-8±1.48E-7	5.92E-8±3.46E-7	<0.001
					K12975	KDO II ethanolaminephosphotransferase	6.14E-8±1.57E-7	6.97E-8±3.55E-7	<0.001
					K12979	Beta-hydroxylase	1.18E-6±3.64E-6	7.99E-7±9.17E-7	0.018
ko03020	RNA polymerase	0.0052±0.0006	0.0054±0.0009	<0.001					
ko00760	Nicotinate and nicotinamide metabolism	0.0114±0.0004	0.0116±0.0005	<0.001	K00322	NAD(P) transhydrogenase	7.37E-7±3.50E-6	4.12E-7±7.85E-7	0.012
					K01799	Maleate isomerase	2.68E-6±2.61E-6	2.45E-6±2.57E-6	0.014
					K08723	5'-nucleotidase	5.41E-8±1.48E-7	5.92E-8±3.46E-7	<0.001
					K13995	maleamate amidohydrolase	1.86E-7±2.72E-7	1.52E-7±2.44E-7	0.001
					K14974	6-hydroxynicotinate 3-monooxygenase	1.86E-7±2.76E-7	1.52E-7±2.44E-7	0.002
					K15357	N-formylmaleamate deformylase	1.89E-7±2.76E-7	1.58E-7±2.53E-7	0.002
					K18028	2,5-dihydroxypyridine 5,6-	1.93E-7±2.83E-7	1.61E-7±2.58E-7	0.002

						dioxygenase			
ko03440	Homologous recombination	0.0167±0.0005	0.0169±0.0005	<0.001	K02317	DNA replication protein DnaT	5.38E-8±1.48E-7	5.90E-8±3.46E-7	<0.001
					K02342	DNA polymerase III subunit epsilon	0.002±0.00007	0.0011±0.00009	0.002
					K02345	DNA polymerase III subunit theta	5.94E-8±1.61E-7	7.12E-8±4.13E-7	<0.001
					K03655	ATP-dependent DNA helicase RecG	0.0011±0.00009	0.0011±0.00015	0.005
ko00121	Secondary bile acid biosynthesis	0.00027±0.0030	0.00015±0.0018	<0.001	K01442	Choloylglycine hydrolase	9.33E-6±0.0001	5.17E-6±0.00006	<0.001
ko04144	Endocytosis	4.79E-6±0.00001	4.09E-6±0.00001	0.019	K12472	Epidermal growth factor receptor substrate 15	0.00001±0.00003	0.00001±0.00003	0.016
ko05111	Vibriocholerea pathogenic cycle	0.0027±0.00028	0.0022±0.0010	<0.001					
ko00621	Dioxin degradation	0.0016±0.0020	0.0010±0.0017	<0.001	K00462	Biphenyl-2,3-diol 1,2-dioxygenase	7.57E-7±7.77E-7	6.62E-7±7.10E-7	0.012
					K00480	salicylate hydroxylase	3.46E-6±3.56E-6	3.14E-6±3.29E-6	0.027
					K01617	2-oxo-3-hexenedioate decarboxylase	1.51E-6±1.63E-6	1.28E-6±1.34E-6	0.009
					K01666	4-hydroxy 2-oxovalerate aldolase	2.14E-7±2.33E-6	5.64E-8±1.85E-7	0.005
					K04073	Acetaldehyde dehydrogenase	5.58E-8±1.36E-7	3.45E-8±1.16E-7	0.004
					K18364	2-oxopent-4-enoate	5.92E-7±7.28E-7	4.88E-7±5.08E-7	0.012
					K18365	4-hydroxy-2-oxovalerate	6.01E-7±7.78E-7	4.89E-7±5.09E-7	0.012
					K18366	Propanal dehydrogenase	6.00E-7±7.78E-7	4.89E-7±5.09E-7	0.012
ko00460	Cyanoamino acid metabolism	0.0011±0.0024	0.00053±0.0017	<0.001	K01455	Formamidase	0.000012±0.00009	2.16E-6±7.38E-6	<0.001
					K13051	L-asparaginase	2.19E-6±2.23E-6	1.96E-6±2.06E-6	0.033
ko00410	beta-Alanine metabolism	0.0023±0.0026	0.0015±0.024	0.002	K00137	Aminobutyraldehyde dehydrogenase	1.24E-7±4.03E-7	1.16E-7±4.02E-7	0.004

					K01580	Glutamate decarboxylase	2.65E-7±5.68E-7	2.76E-7±9.81E-7	0.002
					K01825	3-hydroxyacyl-CoA dehydrogenase	6.93E-7±3.46E-7	3.67E-7±7.55E-7	0.005
					K17722	dihydropyrimidine dehydrogenase (NAD+) subunit PreT	6.71E-8±1.73E-7	9.23E-8±3.45E-7	0.011
					K17723	Dihydropyrimidine dehydrogenase (NAD+) subunit PreA	9.61E-7±9.66E-7	9.51E-7±9.83E-7	3.11E-01
ko00623	Toluene degradation	0.0032±0.0031	0.0024±0.0030	0.012	K00055	Aryl-alcohol dehydrogenase	5.47E-7±7.96E-6	9.66E-8±5.63E-7	<0.001
					K00141	Benzaldehyde dehydrogenase (NAD)	2.43E-6±2.36E-6	2.02E-6±2.22E-6	0.044
					K01856	Muconate cycloisomerase	2.50E-6±4.32E-6	2.02E-6±2.11E-6	0.022
					K03381	catechol 1,2-dioxygenase	2.06E-6±4.16E-6	1.61E-6±1.73E-6	0.009
					K05797	4-cresol dehydrogenase (hydroxylating) flavoprotein subunit	6.28E-7±7.96E-6	1.27E-9±1.27E-8	0.036
					K07546	E-phenylitaconyl-CoA hydratase	1.34E-7±1.97E-7	1.12E-7±1.95E-7	0.019
					K15760	Toluene monooxygenase system protein A	4.74E-7±4.39E-7	4.36E-7±4.41E-7	0.038
					K15761	Toluene monooxygenase system protein B	4.74E-7±4.39E-7	4.35E-7±4.41E-7	0.038
					K15762	Toluene monooxygenase system ferredoxin subunit	4.74E-7±4.39E-7	4.36E-7±4.41E-7	0.038
					K15763	Toluene monooxygenase system protein D	4.74E-7±4.39E-7	4.36E-7±4.41E-7	0.038
					K15764	Toluene monooxygenase system protein E	4.74E-7±4.39E-7	4.36E-7±4.41E-7	0.038
					K15765	Toluene monooxygenase electron transfer component	4.74E-7±4.39E-7	4.36E-7±4.41E-7	0.038
					K16242	Phenol/toluene 2-monooxygenase (NADH) P3/A3	6.14E-7±1.66E-6	4.74E-7±5.05E-7	0.031
					K16243	Phenol/toluene 2-monooxygenase (NADH) P1/A1	5.12E-7±4.96E-7	4.59E-7±4.72E-7	0.022
					K16244	Phenol/toluene 2-monooxygenase (NADH) P2/A2	5.12E-7±4.96E-7	4.59E-7±4.72E-7	0.022
					K16245	Phenol/toluene 2-monooxygenase (NADH) P4/A4	6.14E-7±1.66E-6	4.74E-7±5.05E-7	0.031
					K16246	Phenol/toluene 2-monooxygenase (NADH) P5/A5	6.15E-7±1.67E-6	4.74E-7±5.05E-7	0.028

					K16249	Phenol/toluene 2-monooxygenase (NADH) P0/A0	5.12E-7±4.96E-7	4.58E-7±4.71E-7	0.021
ko00791	Atrazine degradation	0.0028±0.0058	0.0016±0.0046	0.007	K01941	Urea carboxylase	8.96E-7±2.39E-6	6.25E-7±8.56E-7	0.003
					K03383	Cyanuric acid amidohydrolase	2.48E-8±6.53E-8	2.06E-8±8.03E-8	0.013

*p-values were obtained from Wilcoxon Rank-Sum Test

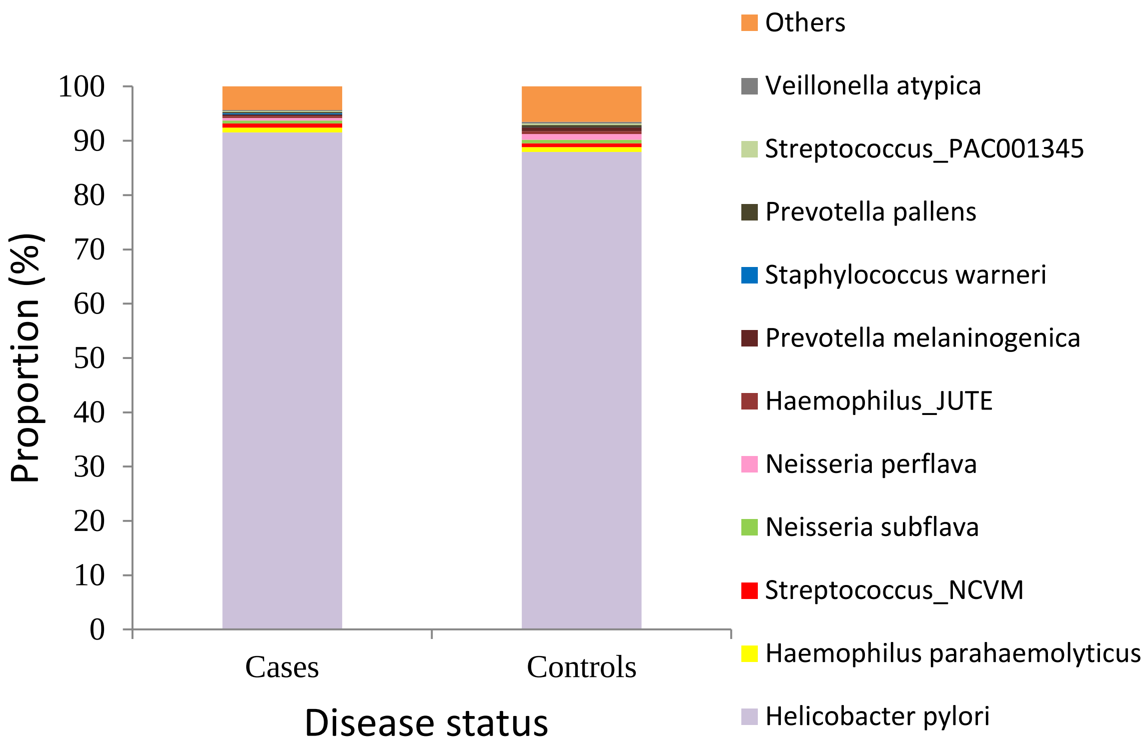

Figure S1. Bar plot of the taxonomic profiles between GC cases and controls in species level.

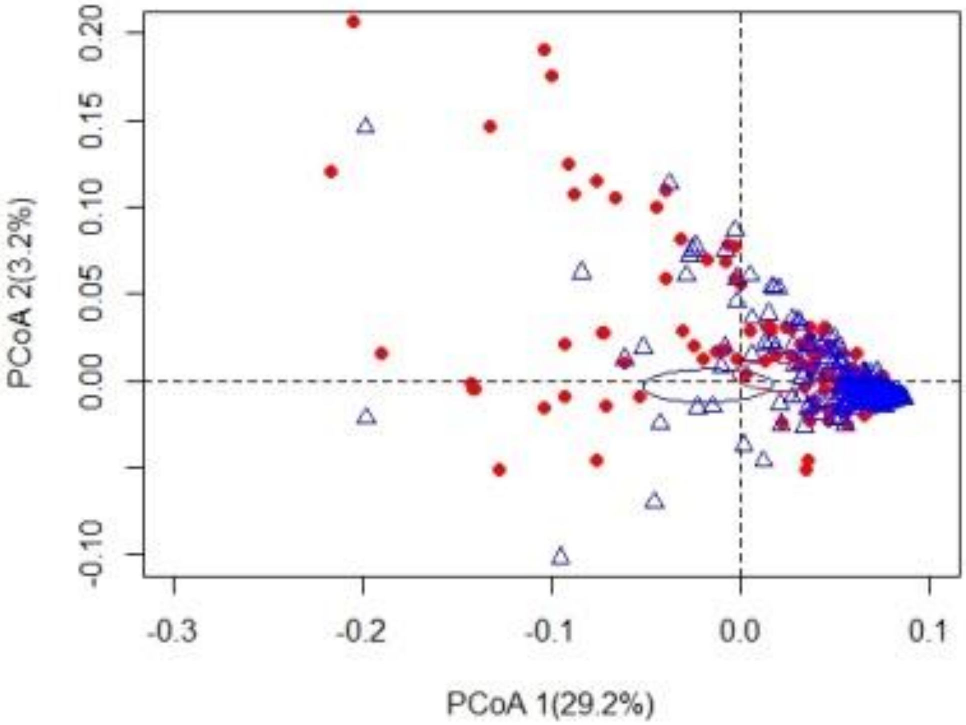

Figure S2. Principal coordinate analysis (PCoA) plot of the Bray-Curtis distance. The red dots indicate the cases, while the blue triangles indicate the controls. The red and blue ellipses represent where 95% of the data belong to controls and cases, respectively, at 5% significance.
